# Breast carcinoma tumour-infiltrating lymphocytes in pre- and post-systemic therapy in HIV-positive and HIV-negative women

**DOI:** 10.4102/sajhivmed.v26i1.1741

**Published:** 2025-10-17

**Authors:** Mishka Adam, Jenny Edge, Louis J. de Jager

**Affiliations:** 1 Division of Anatomical Pathology, Faculty of Medicine and Health Sciences, Stellenbosch University, Cape Town, South Africa; 2 Division of Anatomical Pathology, Tygerberg Hospital, National Health Laboratory Service, Cape Town, South Africa; 3 Department of Surgery, Faculty of Medicine and Health Sciences, University of the Witwatersrand, Johannesburg, South Africa; 4 Division of Anatomical Pathology, PathCare, Cape Town, South Africa

**Keywords:** HIV, women with breast carcinoma, TILs, South Africa, RCB index

## Abstract

**Background:**

HIV-positive women with breast cancer do not exhibit significant differences in tumour characteristics when compared to their HIV-negative counterparts. Stromal tumour-infiltrating lymphocytes (TILs) serve as an important indicator of the host’s capacity to combat malignancy, particularly during the early stages of tumour progression.

**Objectives:**

The objective of this study was to assess and compare the pathological characteristics of breast carcinomas, specifically focusing on TILs in histological specimens obtained before and after systemic therapy, between HIV-positive and HIV-negative patient groups at a public hospital in the Western Cape province. Additionally, the study aimed to determine whether a higher percentage of TILs was associated with a favourable treatment response.

**Method:**

A retrospective cohort study was conducted, incorporating a negative control group matched for histological subtype, and intrinsic subtypes among patients diagnosed between January 2017 and December 2018.

**Results:**

There was no significant difference in TILs before and after treatment, nor was there a difference between patients treated with neoadjuvant chemotherapy (NACT) compared to those receiving endocrine therapy (ET) within both groups. A complete pathological response was achieved in four HIV-positive patients (14%) and one HIV-negative patient (2%). An inversely proportional relationship was noted between TILs and CD4 counts prior to treatment.

**Conclusion:**

This study found no significant differences in TILs between HIV-positive and HIV-negative women with breast cancer. There is a need for further research on the prognostic value of TILs, especially for guiding additional treatment options including the use of immune checkpoint inhibitors.

**What this study adds:** This was the first study that evaluated TILs in breast cancer in HIV-positive and HIV-negative patients, where associations were drawn between TILs percentage at baseline and after surgery, pathological tumour characteristics, association with therapy, as well as with RCB classes. Intermediate to high TILs in residual disease represent the ideal phenotype for possible treatment with immune checkpoint inhibitors as they represent the presence of a reduced antitumour immune response.

## Introduction

Breast cancer is the most common malignancy in women globally and is a leading cause of cancer-related mortality.^1^ South Africa has the largest number of people living with HIV globally.^2^

The immune system is critical for maintaining tissue homeostasis in immunosurveillance and initiating immune responses.^3^ Tumour-infiltrating lymphocytes (TILs) are defined as mononuclear host immune cells present within the boundary of a tumour and are located within the stroma between carcinoma cells, without being in direct contact with or infiltrating the tumour cell nests.^3,4^ Seventy-five per cent of TILs are T-cells. CD8+ T-cells are the group of lymphocytes that correlate best with favourable clinical outcomes and are associated with longer survival rates.^5^

TILs are a useful prognostic and predictive biomarker, especially in triple negative breast cancers (TNBC) which are more common in HIV-positive women (20%) when compared to HIV-negative women (15%).^6,7^ Studies have shown that breast carcinomas are infiltrated by a heterogenous population of T-cells, B-cells, natural killer (NK) cells and macrophages. T-lymphocytes comprise CD4+ helper T-cells and CD8+ cytotoxic T-cells. Antitumour effects are mediated by the ability of CD8+ T-cells to induce a response against a specific antigen.^8^ There are three phases of the so-called immunoediting process which allows tumour cells to escape and eventually be eliminated from immune control: elimination, equilibrium, and the escape phase.^5,9^ Patients typically present and are diagnosed once the tumour has reached a stage that allows it to evade immunosurveillance, resulting in symptoms or palpable masses.^5,10^

An increased density of TILs predicts a response to neoadjuvant chemotherapy in all intrinsic subtypes of breast carcinoma.^11,12^ TILs are most commonly found in the highly proliferative TNBC and HER2-positive breast cancers, where an association between increased TILs and improved survival outcomes in these two intrinsic subtypes has been shown.^3,11,12,13^ The presence of TILs in both TNBC and HER2-enriched cancers are independent indicators of better outcome regarding disease-free survival, distant recurrence-free survival and overall survival and better response to both standard chemotherapy and trastuzumab.^3,14,15,16^

The newer immune checkpoint inhibitors have been shown to improve progression-free survival in metastatic TNBC with high PD-L1 expression, and tumours with a high amount of TILs demonstrate increased PD-L1 expression.^11,15,17^ This could explain why TNBC have shown a greater response to immune checkpoint inhibitors.^11^ The presence of TILs after neoadjuvant chemotherapy is a positive prognostic indicator and is associated with improved survival rates.^5,18,19^ In contrast, increased TILs were identified as an adverse prognostic factor for survival in hormone receptor-positive, HER2-negative breast cancers, suggesting a distinct biological pathway for the immunological infiltrate in this subtype.^11^

The Residual Cancer Burden (RCB) index after neoadjuvant therapy is a prognostic factor for local and distant relapse. The pCR (complete pathological response) category, defined by the absence of invasive tumour in the breast and lymph nodes after neoadjuvant therapy, serves as a surrogate predictor of long-term survival.^20,21,22^

The aim of this retrospective cohort study was to classify and characterise the clinicopathological features of breast carcinoma in HIV-positive women in comparison with an HIV-negative grouped matched according to histological subtype. The variables recorded and analysed in this study included age at diagnosis, histological grade, intrinsic subtypes, and the presence or absence of treatment. The relationship between the baseline TILs percentage and clinicopathological variables was assessed. The primary outcome of interest was the achievement of pCR, as determined by the evaluation of the resection specimen following systemic therapy.

## Research methods and design

### Study design

This study was a retrospective cohort analysis utilising data and histological slides retrieved from the archives of the Division of Anatomical Pathology at the National Health Laboratory Services at Tygerberg Hospital, Cape Town, South Africa, focusing on women diagnosed with breast cancer between 2017 and 2018.

### Study sample

Two patient groups were analysed in this study. In the HIV-positive cohort, the variables assessed included patient age, histological and intrinsic subtype, as well as the RCB-class and index. All variables were compared with an HIV-negative control group, which consisted of patients diagnosed within the same time frame and matched according to histological subtypes.

### Data collection

**FIGURE 1:**
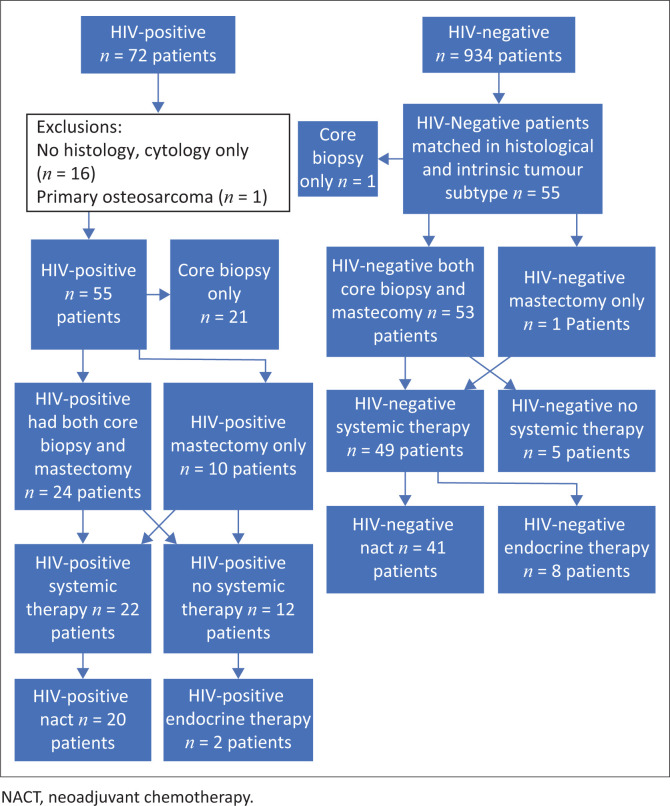
Cohort patient flow. An indication of the patients in this study that were included in assessing tumour-infiltrating lymphocytes data and tumour characteristics.

A total of 72 HIV-positive patients were identified among 1006 patients during this period. Histology was available for 55 of the 72 HIV-positive patients (Figure 1). This included cases with both core biopsies and mastectomies, core biopsies alone, or mastectomies performed based on fine needle aspiration findings. All relevant variables were evaluated and documented for patients with available histology. However, pre-treatment TILs could not be evaluated in cases where core biopsies were not performed. Histologically confirmed breast cancer slides from both HIV-positive and HIV-negative patients were retrieved for evaluation, including haematoxylin and eosin sections and immunohistochemical (IHC) stains. The following variables were analysed. TILs were assessed in pre- and post-treatment specimens, including core biopsies and mastectomies. The tumour was subtyped histologically and graded according to the Nottingham system for both diagnostic and post-treatment specimens. The presence and grade of ductal carcinoma *in situ* (DCIS), if present, were recorded in both diagnostic and post-treatment specimens. CD4 counts in the HIV-positive group were recorded. The tumours were classified into intrinsic subtype categories according to their IHC profiles (Luminal A, Luminal B, HER2-enriched, and TNBC), which were determined based on IHC staining patterns and *in situ*-hybridisation for equivocal HER2 IHC staining. The RCB indices and classes were calculated using the RCB Calculator (https://www3.mdanderson.org/app/medcalc/index.cfm?pagename=jsconvert3).

### Evaluation of stromal TILs

Stromal TILs were classified according to the recommendations by the Immuno-Oncology Biomarker Working Group.^12,19^ They were analysed by two pathologists using microscopic valuation. TILs were recorded as a continuous variable and categorised into three distinct groups: low (0% – 15%), intermediate (15% – 60%), and high (> 60%). Only stromal TILs were evaluated, excluding intratumoral lymphocytes. The entire tumour bed was systematically evaluated at low magnification (x20, with a 1 mm field diameter, and x40, with a 0.5 mm field diameter), allowing for a comprehensive assessment of the tissue architecture. All available slides containing tumour bed from both the core biopsies and mastectomy specimens were thoroughly examined to calculate the average percentage of TILs across the entire tumour area. In instances where a pCR was observed, post-treatment assessment of TILs was not conducted.

### Statistical analysis

The R software environment was employed for statistical computing and data visualisation. The study evaluated the association between the percentage of TILs at baseline and the percentage at the time of residual disease (RD). Clinicopathological variables included age categories (< 40, 40–60, and > 60 years), histological grade (I, II, or III), intrinsic subtypes, and the presence or absence of treatment (neoadjuvant chemotherapy, endocrine therapy [ET], or no therapy). The relationship between the baseline TIL percentage and clinicopathological variables was assessed. The primary outcome of interest was the achievement of a pCR, as determined by the evaluation of the resection specimen following systemic therapy. In cases where pCR was achieved, TILs were not assessed in the post-treatment specimens. The analysis aimed to ascertain whether a high percentage of TILs (> 60%) could serve as a predictor for a favourable outcome (pCR). TILs were categorised into three groups: low (0% – 15%), intermediate (15% – 60%), and high (> 60%).

### Statistical approach

Data visualisation in the form of scatter and box plots of the relationships between TILs and the other variables such as RCB-index, RCB-class, intrinsic subtype, and CD4 count was done.

The Welch Two Sample *t*-test with unequal variances was implemented to establish differences in mean TILs between: HIV-positive and HIV-negative patients, patients treated with neoadjuvant chemotherapy and ET. The alpha level of statistical significance was 5%.

### Ethical considerations

All patient samples selected to be part of this study were de-identified and assigned with a new unique case number to ensure anonymity. Only the principal investigator and supervisors had access to the data. This project was reviewed by members of the Health Research Ethics Committee (HREC) of Stellenbosch University (project ID: 23065; HREC reference number: S21/07/119). A waiver of consent was requested and granted by the HREC committee as there was no risk to the participants. This study was performed in accordance with Good Clinical Practice and the Declaration of Helsinki.

## Results

### Patient and tumour characteristics

The study included a total of 110 participants, comprising 55 individuals (50%) in the HIV-positive group and 55 individuals (50%) in the HIV-negative group. Both groups met the inclusion criteria and were matched based on histological subtype. Participants were further categorised according to intrinsic subtypes, with all individuals diagnosed within the same time frame.

Among the study participants, 22 patients underwent core biopsies exclusively, while 11 patients underwent mastectomies exclusively based on their fine needle aspiration results. Ten patients in the HIV-positive groups had mastectomies without core biopsies, and pre-treatment TILs could not be assessed in these cases. Tumour variables and TILs were recorded in these cases that had mastectomies only; however, no statistical correlation of TILs and RCB indices pre- and post-treatment could be determined.

Table 1 compares variables and tumour characteristics between the HIV-positive and HIV-negative groups. HIV-positive women tended to be younger, with a higher proportion aged under 40 years. There were differences in tumour grades between the two groups. The HIV-positive cohort had more patients with Grade III tumours (20%) than the HIV-negative group (9%). Fewer HIV-positive women received systemic therapy in comparison with the HIV-negative group.

**TABLE 1:** Comparison of variables and tumour characteristics between the HIV-positive and HIV-negative groups.

Variable	HIV status			
	HIV-positive		HIV-negative	
	*n*	%	*n*	%
**Age (years)**	**55**	**100**	**55**	**100**
< 40	11	20	9	16
40–60	39	71	30	55
> 60	5	9	16	29
**Histology available (core biopsy, resection, or both)**	**55**	**100**	**55**	**100**
Core biopsy and mastectomy	24	44	53	96
Core biopsy only	21	38	1	2
Mastectomy only	10	18	1	2
**Tumour grade**	**55**	**100**	**55**	**100**
Grade I	7	13	8	15
Grade II	37	67	42	76
Grade III	11	20	5	9
**TILs**				
**Pre-treatment TILs**	**45**	**100**	**55**	**100**
Low < 15%	21	47	33	60
Intermediate 15% – 60%	18	40	19	35
High > 60%	6	13	3	5
**Post-treatment TILs**	**22**	**100**	**49**	**100**
Low < 15%	8	36	25	51
Intermediate 15% – 60%	6	27	18	37
High > 60%	4	18	5	10
No residual tumour – complete pathologic response	4	18	1	2
**Intrinsic subtype**	**55**	**100**	**55**	**100**
Luminal A	22	40	23	42
Luminal B	11	20	7	13
HER2 enriched	11	20	14	25
Triple negative	8	15	8	15
Could not be determined: no tumour, DCIS only, no IHC stains done	3	5	3	5
**Treatment**	**55**	**100**	**55**	**100**
Chemotherapy	20	36	41	74
Endocrine therapy	2	4	8	15
No therapy	12	22	5	9
Not applicable: core biopsy only, no resection done	21	38	1	2
**RCB-class (post NACT and endocrine therapy)**	**22**	**100**	**49**	**100**
I	2	9	1	2
II	7	32	22	45
III	9	41	25	51
pCR	4	18	1	2

TIL, tumour-infiltrating lymphocyte; DCIS, ductal carcinoma *in situ*; IHC, immunohistochemical; RCB, residual cancer burden; NACT, neoadjuvant chemotherapy; pCR, complete pathological response.

### Comparison of TILs between the HIV-positive and HIV-negative groups

#### T-test applied across whole sample

The output was the result of a Welch Two Sample *t*-test performed in R. The test compared the means of TILs across the two groups; both prior to and post treatment.

The *t*-value of –0.5386 was the test statistic. The Welch Two Sample *t*-test showed that with a *P* = 0.5908 (*P* > 0.05) in Table 2, we concluded in favour of the null hypothesis that there was no statistically significant difference in TILs between HIV-positive and HIV-negative groups.

**TABLE 2:** Summary statistics table for TILs comparing the HIV-positive and HIV-negative groups.

Group	Variable	Mean TILs (%)	*t*	*df*	*P*	95% CI
HIV-positive	Pre-treatment TILs	24.27083	−0.00255	59.892	0.998	−11.67959 to 11.64983
	Post-treatment TILs	24.28571	-	-	-	-
HIV-negative	Pre-treatment	17.07547	−1.1071	103.98	0.2708	−11.32294 to 3.209733
	Post-treatment	21.13208	-	-	-	-
Entire sample (HIV-positive and HIV-negative groups)	Pre-treatment	20.49505	−0.5386	176.4	0.5908	−8.055822 to 4.601477
	Post-treatment	22.22222	-	-	-	-
TILs with NACT in comparison with ET	Post-treatment	-	1.5096	18.976	0.1476	−2.870934 to 17.724735

*df*, degrees of freedom; CI, confidence interval; TIL, tumour-infiltrating lymphocyte; NACT, neoadjuvant chemotherapy; ET, endocrine therapy.

### Association between pre- and post-treatment TILs for the HIV-positive and HIV-negative groups

#### HIV-positive group

The Welch Two Sample *t*-test showed that with a *P* = 0.998 (*P* > 0.05) in Table 2, we concluded in favour of the null hypothesis that there was no statistically significant difference between the mean pre- and post-treatment TILs in the HIV-positive group.

#### HIV-negative group

The Welch Two Sample *t*-test showed that with a *P* = 0.2708 (*P* > 0.05) in Table 2, we concluded in favour of the null hypothesis that there was no statistically significant difference in TILs mean values before and after treatment in the HIV-negative group.

### TILs and RCB classes in patients treated with neoadjuvant therapy in comparison with ET

#### Post-treatment interpretation of TILs

The *t*-value was 1.5096, which meant that the difference in mean TILs values between the NACT and ET groups is 1.5096 standard errors away from zero. The Welch Two Sample *t*-test showed that with a *P* = 0.1476 (*P* > 0.05) in Table 2, we concluded in favour of the null hypothesis that there was no statistically significant difference in mean TILs values between the NACT and ET groups. However, it was possible that a difference between the groups existed that was not detected by this analysis, due to the limited number of patients that received ET.

#### Post-treatment therapy interpretation of RCB

The test results show that the calculated *t*-statistic was −0.79298. The Welch Two Sample *t*-test showed that with a *P* = 0.4378 (*P* > 0.05) in Table 2, we concluded in favour of the null hypothesis that there was no significant difference between the RCB classes for the two groups.

### Association between TILs and CD4 counts in the HIV-positive group

**FIGURE 2:**
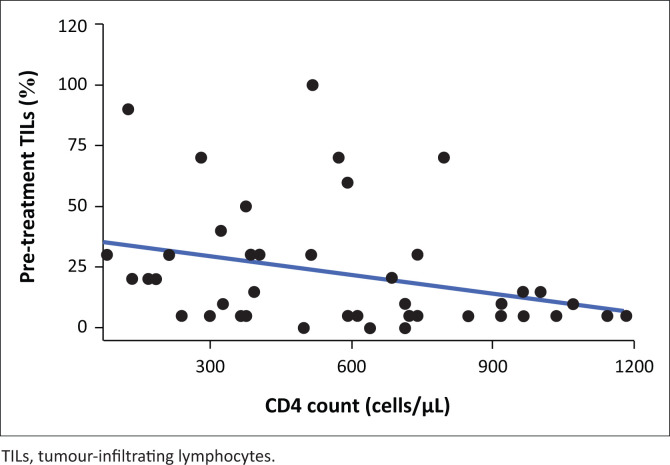
Scatterplot showing the trend between pre-treatment TILs and CD4 count.

**FIGURE 3:**
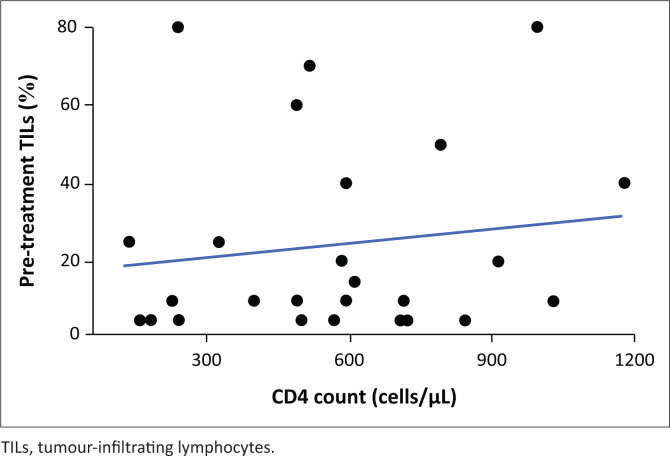
Scatterplot showing the trend between post-treatment TILs and CD4 count.

Figure 2 shows the inverse relationship between CD4 counts and TILs, indicating that pre-treatment TILs decrease with an increase in CD4 count. Conversely, Figure 3 illustrates a positive relationship between CD4 counts and post-treatment TILs, showing that post-treatment TILs are higher with an increase in CD4 count.

### Association between pre-treatment TILs and RCB classes

**FIGURE 4:**
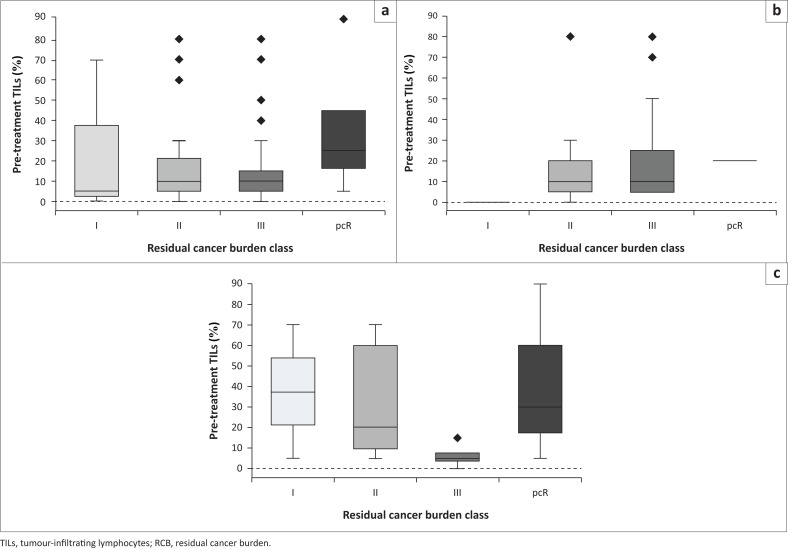
Boxplots showing the distribution of pre-treatment TILs versus RCB classes for (a) all patients, (b) HIV-negative patients, and (c) HIV-positive patients.

The box plots in Figure 4 show that those patients with a low RCB class had a wider range of pre-treatment TILS. A higher RCB class was associated with a narrower range of pre-treatment TILs, with values clustering at the lower end of the TIL measurement scale. In the HIV-negative group, a higher RCB class was characterised by an increased amount of pre-treatment TILs. In contrast, in the HIV-positive group, a higher quantity of pre-treatment TILs was observed in RCB-class I (RCB-I).

### RCB classes

In Table 1, the RCB within the HIV-positive group was 9% for class I, 32% for class II, and 41% for class III, with 18% (*n* = 4) of patients achieving pCR. In the HIV-negative group, the RCB was 2% for class I, 45% for class II, and 51% for class III, with only 2% (*n* = 1) of patients achieving pCR.

All patients that achieved pCR after treatment had received neoadjuvant chemotherapy. Although the sample size was small, in the HIV-positive group, one patient had only a mastectomy specimen available, and no core biopsy was available to assess for pre-treatment TILs. Additionally, no patients that had received ET achieved pCR in this sample.

### Intrinsic subtypes and TILs

**FIGURE 5:**
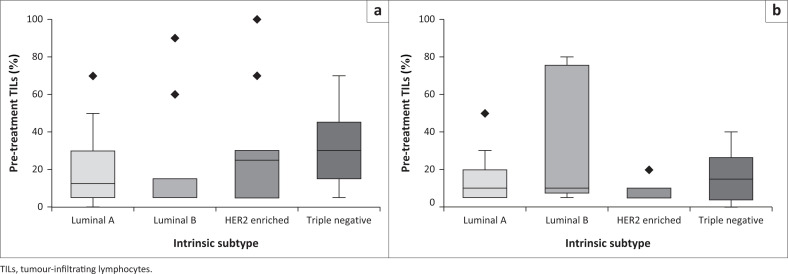
Boxplots depicting the distribution of TILs versus intrinsic subtypes in the (a) HIV-positive and (b) HIV-negative groups.

In Figure 5, the TNBC subtype demonstrated higher median TILs in comparison to the other intrinsic subtypes in both groups. Two of the patients that achieved pCR were Luminal B subtype, and only one was a TNBC. No association between pCR and TILs in different intrinsic subtypes could be determined because of the small number of patients that achieved pCR.

## Discussion

To our knowledge, this was the first study that evaluated TILs in breast cancer in HIV-positive and HIV-negative patients, where associations were evaluated between TILs percentage at baseline and after surgery, pathological tumour characteristics, association with NACT and ET, as well as with RCB classes. The distribution of age between the two groups revealed differences between the two groups. The HIV-positive group were younger overall, with 71% aged 40–60 years, compared to 55% in the HIV-negative group. Seventy-four per cent of HIV-negative patients received NACT compared to HIV-positive patients (36%). In contrast, a higher percentage of HIV-positive patients did not receive therapy (22%) compared to HIV-negative patients (9%). This may be accounted for as the groups were not matched for stage.

Tumour grade distribution also differed between groups. HIV-positive patients had a slightly lower proportion of Grade II tumours (67%) compared to HIV-negative patients (76%), but a higher proportion of Grade III tumours (20% vs. 9%). The most common tumour types in both the HIV-positive and HIV-negative groups were invasive breast carcinoma of no special type. Most patients across both groups had Grade II tumours, although the HIV-positive group did have a slightly higher proportion of Grade III tumours (20%) in comparison with the HIV-negative group (9%). Twenty-two (40%) of patients in the HIV-positive group received treatment, either in the form of NACT or ET. Forty-nine (89%) patients in the HIV-negative group received treatment.

We hypothesised that HIV-negative patients with breast carcinoma would have a larger number of stromal TILs than the HIV-positive group, and that a higher baseline lymphocytic infiltrate would be associated with a better pathological response than those with a lower number of TILs. However, there was only one case in the HIV-positive group that had a high number of pre-treatment TILs that achieved pCR, which was a Luminal B, HER2-negative tumour. One TNBC intrinsic subtype in the HIV-positive group achieved pCR, but had a low number of TILs on the core biopsy specimen. Within the HIV-negative group, there was only one patient that achieved pCR that had an intermediate level of TILs. The outcome of pCR was observed in too few samples for associations to be determined, as they demonstrated variation in TILs and different intrinsic subtypes. Since two patients in this study who achieved a complete pCR had undergone mastectomies without prior core biopsies, pre-treatment TILs could not be assessed and no association between high TILs and pCR could be established.

Within the HIV-positive group, four patients achieved pCR with no clear correlation to pre-systemic therapy TILs. The results may suggest that the poor prognosis of tumours with high TILs was driven by the larger group of patients that did not have a complete pathological response. In the pre-treatment cohort, an inverse relationship was observed between TILs and CD4 counts, suggesting that lower CD4 counts were associated with fewer TILs. This contrasts with the post-treatment findings, where CD4 counts and TILs levels exhibited a positive correlation, indicating that systemic therapy might have enhanced the immune response. The RCB index represents a novel and independent risk factor that enhances the prediction of distant relapse following neoadjuvant chemotherapy. Studies have shown that pathologic response to NACT is prognostic in all phenotypic subsets of cancer.^21^ The utilisation of RCB adds prognostic information in cases where pCR is not achieved. Evaluating the RCB indices and classes offers valuable long-term prognostic information and can aid in making informed decisions regarding adjuvant therapy.^22^

There were three possible scenarios of TILs in RD post-treatment: intermediate to high TILs in RD, low TILs in RD, and a change in TILs from before to after systemic therapy. The first scenario represents the ideal phenotype for immune checkpoint therapy as TILs represent the presence of, but partially reduced, antitumour immunity, which only needs to be augmented or maintained.^19^

The results of this study highlighted the variation in pre- and post-treatment TILs in both the HIV-negative and HIV-positive groups. No association between TILs pre- and post-systemic therapy was found where residual invasive disease was present across both groups. In this study, however, only one patient in the HIV-positive group achieved pCR that had a TNBC subtype out of a total of five pCR patients across the whole sample.

Assessment of TILs by digital analysis and machine learning algorithms is possible and may be useful for standardisation as tumour heterogeneity contributes to interobserver variability. However, this may not be possible in resource-limited settings, where there may be little or no access to slide scanners and the necessary software.^23^ The ring studies identified intratumoural heterogeneity as a major limiting factor in assessing TILs and attempted to address these constraints and improve concordance, which is a step towards improved standardisation of reporting tumour biological parameters.^24^ The use of IHC staining to identify and subtype the immune cells comprising TILs is not routinely performed, and requires additional staining and further standardisation techniques.^12,19^ This may be useful, as CD8+ T-cells have been shown to be associated with a significant reduction in the relative risk of death caused by ER-negative breast tumours.^25^

The strengths of this study included the assessment of TILs using consensus guidelines,^12^ the use of predefined cutoff points and methods, and a low degree of interobserver variability, and was the first study of its kind assessing TILs in breast cancer in HIV-positive women. Interobserver variability was low, as the entire tumour bed area was assessed and an average was then determined, as reported in the ring 2 study where at least three separate areas of tumour were assessed.^24^ Another advantage of this study was that TILs were evaluated in both the core biopsy and excision specimens; this reflects what routinely would be performed in histological analysis of these specimens.

Further research into why some TNBC and HER2-positive breast cancers cannot generate a host antitumour immune response and how trastuzumab alters the immune biological environment is needed. It is not yet known if patients with TILs at RD may benefit from immune checkpoint inhibitors.^26^ Assessing TILs and including this information into pathology reports is a practical approach, particularly in lower-income countries where the cost and feasibility of PD-L1 assays may be restrictive.^27^

### Limitations

The immune cell phenotypes were not analysed in this study. An explanation for the differences between luminal tumours and TNBC may be because of the contribution of different cell types.

The use of antiretroviral therapy (ART) was shown to not affect the clinicopathological characteristics of breast cancer in an observational study.^28^ We did not have information on ART use for most of the HIV-positive patients included in this study. For patients on ART, it is difficult to ascertain whether an association between tumour characteristics can be determined, because of several unknown variables. We did not know the duration of their disease, length of treatment, presence or absence of other comorbidities and immunosuppressive conditions, treatment compliance, and whether they had regular follow-up visits to their primary health care facilities for management. To date, the only association between CD4 count and the relative risk of developing a malignancy with an established viral pathogenesis is with the known AIDS-defining malignancies.^2^

We recorded TILs as a continuous variable and then used predefined cutoff values to categorise TILs into three groups. We acknowledge that there are no validated cutoff points for high or low TILs percentages, although Denkert et al. used a cutoff value of greater than 60% for a classification of high TILs.^11^ These cutoff values are arbitrary, as TILs can range from any value between 0% and 100%. Subdivision into various groups may be of value in future trials, as stratification can more easily be done on categorical variables. In this study we have presented categorical and continuous analysis of both approaches, and similar results were seen in both groups.

This study had a small sample size and retrospective design. The limited number of patients that achieved pCR impeded robust statistical analysis, including the assessment of whether higher pre-treatment levels of TILs were predictive of improved outcomes and a pCR. The small sample size also limited the generalisability of the findings and underscores the need for larger, prospective studies to validate these results and explore these associations more thoroughly.

## Conclusion

The findings of this retrospective cohort study revealed no statistically significant differences in TILs between HIV-positive women with breast carcinoma and their HIV-negative counterparts. Although the conclusions drawn from this study were limited by the sample size, it represents the first investigation into the associations between TILs in breast cancer among HIV-positive and HIV-negative women. Additionally, we assessed RCB indices and classes, which aids in providing valuable long-term prognostic information alongside the clinicopathological data and staging. Further exploration to determine the prognostic value of TILs in cases treated with ET are needed. Integrating TILs into pathological reports may improve the identification of patients who are likely to benefit from additional treatment regimens, particularly regarding immune checkpoint inhibitors.
